# Cancer Internet Search Activity on a Major Search Engine, United States 2001-2003

**DOI:** 10.2196/jmir.7.3.e36

**Published:** 2005-07-01

**Authors:** Crystale Purvis Cooper, Kenneth P Mallon, Steven Leadbetter, Lori A Pollack, Lucy A Peipins

**Affiliations:** ^2^Yahoo! Inc (at the time of this study)Dynamic Logic (at present); ^1^Division of Cancer Prevention and ControlNational Center for Chronic Disease Prevention and Health PromotionCenters for Disease Control and PreventionAtlantaGAUSA

**Keywords:** Internet, neoplasms, health education

## Abstract

**Background:**

To locate online health information, Internet users typically use a search engine, such as Yahoo! or Google. We studied Yahoo! search activity related to the 23 most common cancers in the United States.

**Objective:**

The objective was to test three potential correlates of Yahoo! cancer search activity—estimated cancer incidence, estimated cancer mortality, and the volume of cancer news coverage—and to study the periodicity of and peaks in Yahoo! cancer search activity.

**Methods:**

Yahoo! cancer search activity was obtained from a proprietary database called the Yahoo! Buzz Index. The American Cancer Society's estimates of cancer incidence and mortality were used. News reports associated with specific cancer types were identified using the LexisNexis “US News” database, which includes more than 400 national and regional newspapers and a variety of newswire services.

**Results:**

The Yahoo! search activity associated with specific cancers correlated with their estimated incidence (Spearman rank correlation, ρ = 0.50, *P* = .015), estimated mortality (ρ = 0.66, *P* = .001), and volume of related news coverage (ρ = 0.88, *P* < .001). Yahoo! cancer search activity tended to be higher on weekdays and during national cancer awareness months but lower during summer months; cancer news coverage also tended to follow these trends. Sharp increases in Yahoo! search activity scores from one day to the next appeared to be associated with increases in relevant news coverage.

**Conclusions:**

Media coverage appears to play a powerful role in prompting online searches for cancer information. Internet search activity offers an innovative tool for passive surveillance of health information–seeking behavior.

## Introduction

Health care providers [1-3] and their patients [4-7] regularly search for health information online. Internet users generally begin looking for health information using a search engine [8-12], an Internet tool that searches for Web pages containing terms specified by users [13]. In February 2004, Google and Yahoo! were the most popular search engines in the United States, with 38% and 32% of market share, respectively [14].

To date, few studies of Internet search activity related to health topics have been published. Most analyzed the proportion of health and non-health searches on various search engines and found that health searches constituted a small percentage of total searches [15-18]. This finding is not surprising given how infrequently Internet users search for health information compared with how often they look for news reports, product information, and other topics [19]. In any case, a small percentage of total Internet searches translates into thousands of health searches each day [16], and an estimated 95 million Americans have used the Internet at least once to search for health information [20].

Cancer appears to be a health topic of some interest to Internet users. Eysenbach and Köhler [16] found that searches for cancer information accounted for 5% of health-related searches on the search engine MetaCrawler. Phillipov and Phillips [18] found that “skin cancer” was one of only 17 health-related search terms among the most popular 300 Internet keywords identified by Wordtracker, a private research company. Bader and Theofanos [21] studied cancer searches conducted using the search engine AskJeeves during a 3-month period and found the most commonly searched cancers were digestive/gastrointestinal/bowel, breast, and skin. This study also compared the incidence of selected cancers with their associated search activity. While this relationship was not statistically tested, the authors observed that some rarer cancers constituted a higher proportion of total searches than their proportion of total cancer incidence. In addition, Bader and Theofanos, as well as Phillipov and Phillips, noted that media coverage appeared to prompt Internet searches for health topics, but they did not systematically investigate or test the relationship.

The study reported here builds on this prior work by analyzing cancer-related searches conducted in the United States from 2001 to 2003 using the search engine Yahoo! Specifically, we investigated three potential correlates of Yahoo! cancer search activity—estimated cancer incidence, estimated cancer mortality, and the volume of cancer news coverage. Cancers that afflicted more individuals, claimed more lives, and generated more news coverage were expected to be associated with more Internet search activity than other cancers, given the interest generated by relevance and publicity. In addition, we assessed the periodicity of Yahoo! cancer search activity and examined sharp increases in Yahoo! search activity related to specific cancer types.

## Methods

This analysis included three types of 2001–2003 US data: Yahoo! cancer search activity, cancer burden (estimated incidence and mortality), and cancer news coverage. The study protocol was reviewed by the Institutional Review Board of the National Center for Chronic Disease Prevention and Health Promotion and was designated as “research not involving human subjects.”

### Yahoo! Cancer Search Activity

During 2001 (the beginning of the study period), Yahoo! was the most popular US search engine, with a 49% market share; however, in 2003 (the end of the study period), Google surpassed Yahoo! as the leading US search engine [22]. Yahoo! remains a widely used search engine; more than 45 million US Internet users conducted Yahoo! searches in February 2004 [14].

Yahoo! maintains a database of search activity called the Yahoo! Buzz Index [23]. This index provides a search activity score for individual search terms—the words or characters that users type into the Yahoo! search box. Each point of a Yahoo! Buzz Index score equals 0.001% of users searching Yahoo! during the time period of interest (day, week, or month). For example, if 250 out of a total of 1 million users searched for “breast cancer” on January 1, 2001, the Yahoo! “breast cancer” search activity score on this day would be 25 (250/1 million × 100000). For a search term to register a search activity score, it must generate at least 50 searches during the time period for which the score is calculated. Yahoo! search activity generated by search terms can be segmented by country, US state, or selected US cities. This study used daily US Yahoo! search activity data from January 1, 2001 (the earliest date for which Yahoo! search activity data are available) to December 31, 2003. We limited our analysis to Yahoo! searches because at the time of this study no other Internet search engine offered a dataset like the Yahoo! Buzz Index, which provides search activity scores adjusted for the size of the population searching for online information.

Yahoo! employs professional “surfers” or content indexers who manually classify Web pages into one of more than 2000 content categories, such as “movies,” “footwear,” “astrology,” or “cancer or neoplasms.” The Yahoo! Buzz Index classifies search terms in the same content category as the first Web page link that a user “clicks” or activates after conducting a search. For instance, if a user entered the search term “colon” and then clicked on a cancer website, “colon” would be classified as a “cancer or neoplasms” search term. If the user clicked on a grammar website, however, “colon” in that instance would be classified as an “education” search term. When a user does not click on a Web page link or when a user clicks on a Web page link that has not been classified, the Yahoo! Buzz Index categorizes the search term using a variety of algorithms that analyze recent content viewed by the user.

To identify commonly used Internet search terms related to specific cancers, we reviewed the search terms classified in the “cancer or neoplasms” category of the Yahoo! Buzz Index, which generated at least 50 searches in any month from January 2001 to December 2003. This search strategy identified 76 unique search terms, of which 23 were included in the analysis (Table 1). The remaining 53 terms were discarded because they did not relate to a specific cancer or included non-English words. Discarded terms included drug names or treatment modalities, such as “chemotherapy” (n = 19); the names of organizations or events, such as “Race for the Cure” (n = 16); general cancer or anatomy terms, such as “oncology” (n = 11); names of celebrities, such as “Gilda Radner” (n = 5); and the carcinogen “DES” (n = 1). Also, “leucemia” (n = 1), the Spanish word for “leukemia,” was discarded because the Yahoo! Buzz Index does not consistently track foreign words, as it excludes search terms that contain non-English characters. For instance, the Yahoo! Buzz Index would not capture a search term with an accent mark, such as “cáncer colorectal” (Spanish for “colorectal cancer”).

### Cancer Burden

The estimated incidence and mortality for 23 cancers during the study period were obtained from annual burden reports published by the American Cancer Society [24-26]. All cancers with at least 7500 new cases in 2001, 2002, or 2003 were included in the analysis (n = 23) whether or not they were associated with Yahoo! search activity.

### Cancer News Coverage

News reports associated with specific cancer types were identified using the LexisNexis “US News” database, which includes more than 400 national and regional newspapers, such as the *Wall Street Journal* and the *Baltimore Sun,* and a variety of newswire services, such as the Associated Press and UPI (United Press International). News reports published from January 1, 2001, to December 31, 2003, related to specific cancer types were found by locating reports with the identified Yahoo! search activity terms (eg, “breast cancer”) in their headlines. In the case of cancers located in the esophagus and oral cavity, for which no Yahoo! search activity terms were associated, the terms “esophageal cancer” and “oral cancer” were used as the headline search terms.

### Analysis

Descriptive statistics were calculated for the Yahoo! search activity score, estimated incidence, estimated morality, and news coverage volume associated with the cancers included in the study. Spearman rank correlations were used to establish the consistency of these variables across the study period, and the data were aggregated. Next, the relationships between Yahoo! search activity and the potential correlates of interest were tested using Spearman rank correlations.

The relationship between Yahoo! search activity and news coverage was further analyzed for the five cancers with the highest daily Yahoo! search activity. The number of news reports published each day was transformed into a categorical variable with four levels (0 news reports, 1–2 news reports, 3–4 news reports, and 5 or more news reports), and one-way analysis of variance (ANOVA) was used to compare mean daily Yahoo! search activity scores at increasing levels of news coverage. To detect possible periodicity effects, Yahoo! search activity data were visually inspected. Three possible periodicity effects were noted: a rise during weekdays (Monday–Friday) compared with weekends; a rise during national cancer awareness months compared with other months; and a decline during summer months (June–August) compared with other months. These possible effects were tested using *t* tests. Finally, the Yahoo! search activity associated with several cancers was marked by sharp increases of 100% or more from one day to the next. For these cancers, we identified the three highest peaks in 2003 Yahoo! search activity and investigated corresponding news events.

## Results

We found Internet search terms generating Yahoo! search activity associated with 21 of the 23 cancers included in the study (Table 1). Of these, 19 cancers were associated with only one Yahoo! search term each. The 2 remaining cancers were associated with two search terms each: cancers of the brain (“brain tumor” and “brain cancer”) and multiple myeloma (“multiple myeloma” and “myeloma”). In these cases, the daily Yahoo! search activity scores associated with each term were summed into a composite score for these cancers.

The highest mean daily Yahoo! search activity scores were generated by breast cancer (mean = 14.37), lung cancer (mean = 9.08), and leukemia (mean = 7.15). Cancers with the highest US 2001–2003 incidences were breast (n = 611300), prostate (n = 608000), and lung (n = 510800). For cancer mortality, lung (n = 469500), colorectal (n = 170400), and breast (n = 120800) cancer were the leading causes of death. Breast cancer (n = 5840), leukemia (n = 2143), and prostate cancer (n = 1822) were associated with the most US news reports from 2001 to 2003. Some cancers, such as leukemia, ovarian, and testicular, appeared to be associated with more Internet search activity than their burden would dictate.

Cancers were ranked by mean daily Yahoo! search activity score, estimated incidence, estimated mortality, and number of related news reports for each year in the study period (2001 to 2003). To explore the consistency of ranks over the study period within each variable, Spearman rank correlations were determined for each pair of years (2001 and 2002, 2002 and 2003, 2001 and 2003). We found statistically significant correlations (*P* < .001) between all year pairs tested (data not shown). Because the ranks associated with these variables were highly consistent from 2001 to 2003, we aggregated the data across the study period.

**Table 1:** Mean daily Yahoo! search activity score (United States, 2001–2003), estimated incidence, estimated mortality, and number of news reports, by cancer

**Cancer**	**Yahoo! Search Terms**		Mean Daily Yahoo! Search Activity Score*(Rank)		**Estimated** **Incidence(Rank)**	**Estimated** **Mortality(Rank)**		**Number of News** **Reports (Rank)**		
Breast	“breast cancer”	14.37 (1)		611300 (1)			120800 (3)		5840 (1)	
Lung	“lung cancer”	9.08 (2)		510800 (3)			469500 (1)		918 (5)	
Leukemia	“leukemia”	7.15 (3)		92900 (10)			65100 (7)		2143 (2)	
Colorectal	“colon cancer”	7.08 (4)		43120 (4)			170400 (2)		617 (6)	
Prostate	“prostate cancer”	6.13 (5)		608000 (2)			90600 (4)		1822 (3)	
Ovary	“ovarian cancer”	3.71 (6)		72100 (13)			42100 (9)		458 (8)	
Lymphoma	“lymphoma”	3.54 (7)		185500 (5)			78100 (6)		480 (7)	
Uterine, cervix	“cervical cancer”	2.53 (8)		38100 (20)			12600 (19)		392 (9)	
Melanoma	“melanoma”	2.25 (9)		159200 (7)			22800 (16)		376 (10)	
Brain	“brain tumor” “brain cancer”	1.52 (10)		52500 (16)			39300 (10)		925 (4)	
Liver	“liver cancer”	0.70 (11)		50100 (17)			42600 (8)		110 (14)	
Testis	“testicular cancer”	0.62 (12)		22300 (23)			1200 (23)		50 (17)	
Pancreas	“pancreatic cancer”	0.23 (13)		90200 (11)			88600 (5)		185 (11)	
Multiple myeloma	“multiple myeloma” “myeloma”	0.11 (14)		43600 (18)			32900 (15)		185 (11)	
Stomach	“stomach cancer”	0.08 (15)		65700 (14)			37300 (13)		50 (17)	
Uterine, corpus	“uterine cancer”	0.012 (16)		117700 (8)			20000 (18)		17 (22)	
Larynx	“throat cancer”	0.012 (16)		28400 (21)			11500 (21)		30 (20)	
Bladder	“bladder cancer”	0.010 (18)		168200 (6)			37500 (12)		118 (13)	
Soft tissue	“sarcoma”	0.009 (19)		25300 (22)			12200 (20)		25 (21)	
Thyroid	“thyroid cancer”	0.002 (20)		62200 (15)			4000 (22)		40 (19)	
Kidney	“kidney cancer”	0.001 (21)		94500 (9)			35600 (14)		77 (15)	
Oral cavity	-	0.000 (22)		86700 (12)			22400 (17)		69 (16)	
Esophagus	-	0.000 (22)		40200 (19)			38100 (11)		13 (23)	

^*^ Each point of a Yahoo! search activity score equals 0.001% of the population searching Yahoo! on any day.

### Correlates of Yahoo! Cancer Search Activity

We tested the relationships between variables by determining Spearman rank correlations between each pair. Statistically significant correlations were found between all variable pairs (Table 2).

**Table 2:** Spearman rank correlations between mean daily Yahoo! search activity score (United States, 2001–2003), estimated incidence, estimated mortality, and number of news reports

	Spearman Rank Correlation*		
	**Mean Daily Yahoo! Search Activity Score**	**Estimated Incidence**	**Estimated Mortality**
Number of news reports	.88^†^	.62^‡^	.74^†^
Estimated mortality	.66^†^	.71^†^	-
Estimated incidence	.50^§^	-	-

^*^ Spearman rank correlations were done on the rankings reported in Table 1.

^†^ *P* ≤ .001

^‡^ *P* = .002

^§^ *P* = .015

The relationship between Yahoo! search activity and its most statistically significant correlate—news coverage—was further analyzed for the five cancers with the highest daily Yahoo! search activity (breast, lung, leukemia, colorectal, and prostate). For these cancers, the number of news reports published each day was transformed into a categorical variable with four levels. The mean daily Yahoo! search activity at each level was compared using ANOVA, and all tests were statistically significant (*P* ≤ .001). For all five cancers, the mean daily search activity score was higher at each increasing level of news coverage (Table 3).

**Table 3:** Mean daily Yahoo! search activity score (United States, 2001–2003), by number of news reports published daily and cancer

**Cancer**	Mean Daily Yahoo! Search Activity Score^*†^(Number of News Reports )			
	**Days With0** **News Reports**	**Days With1–2** **News Reports**	**Days With3–4** **News Reports**	**Days With5+** **News Reports**
Breast	10.09 (81)	11.49 (278)	13.36 (252)	17.27 (484)
Lung	8.27 (633)	10.00 (362)	10.54 (71)	11.71 (29)
Leukemia	6.89 (248)	7.07 (523)	7.18 (232)	8.26 (92)
Colorectal	6.72 (739)	7.44 (297)	8.25 (43)	13.92 (16)
Prostate	5.30 (390)	6.40 (467)	6.72 (150)	7.43 (88)

^*^ Each point of a Yahoo! search activity score equals 0.001% of the population searching Yahoo! on any day.

^†^ ANOVA was used to compare the mean daily Yahoo! search activity at each level of news coverage. For all five cancer sites, a statistically significant difference (*P* ≤ .001) was found.

### Periodicity of Yahoo! Cancer Search Activity and News Coverage

Three possible periodicity effects were tested: a rise during weekdays (Monday–Friday) compared with weekends; a rise during national cancer awareness months compared with other months; and a decline during summer months (June–August) compared with other months. To test for these potential effects, we used the five cancers with the highest daily mean Yahoo! search activity scores (breast, lung, leukemia, colorectal, and prostate) (Table 4). For all five cancers tested, both mean daily Yahoo! search activity scores and mean daily news reports were higher Monday–Friday than they were Saturday–Sunday (*P* < .001). Higher mean daily Yahoo! search activity scores were found for breast cancer (*P* < .001), lung cancer (*P* < .001), and colorectal cancer (*P* < .001) during their respective national awareness months. The number of mean daily news reports related to breast cancer (*P* < .001), colorectal cancer (*P* < .001), and prostate cancer (*P* = .007) rose during their respective national awareness months. Mean daily Yahoo! search activity scores for breast cancer (*P* < .001), lung cancer (*P* < .001), and leukemia (*P* < .001) were lower during the summer months than during the rest of the year. While mean daily news reports about breast cancer also decreased during the summer (*P* < .001), mean daily news reports about prostate cancer rose (*P* = .01).

**Table 4:** Periodicity of mean daily Yahoo! search activity score (United States 2001–2003) and mean daily number of news reports, by cancer

**Cancer**		**Weekdays**	**Weekends**	***P* value**	**Awareness Month**	**Non-Awareness Months**	***P* value**	**Summer: June-August**	**Non-Summer**	***P* value**
Breast	Mean Daily Yahoo! Search Activity Score^*^	15.78	10.84	< .001	26.33	13.26	< .001	10.78	15.58	< .001
	Mean Daily Number of News Reports	6.26	3.02	< .001	15.30	4.41	< .001	4.19	5.72	< .001
Lung	Mean Daily Yahoo! Search Activity Score	10.31	6.00	< .001	11.69	8.84	< .001	5.76	10.20	<.001
	Mean Daily Number of News Reports	1.03	0.37	< .001	1.03	0.82	.226	0.70	0.89	.086
Leukemia	Mean Daily Yahoo! Search Activity Score	8.13	4.70	< .001	6.65	7.20	.093	5.65	7.66	< .001
	Mean Daily Number of News Reports	2.20	1.34	< .001	1.51	2.00	.036	1.88	1.98	.506
Colorectal	Mean Daily Yahoo! Search Activity Score	7.73	5.44	< .001	10.46	6.77	< .001	6.83	7.17	.081
	Mean Daily Number of News Reports	0.68	0.27	< .001	1.55	0.47	< .001	0.49	0.59	.214
Prostate	Mean Daily Yahoo! Search Activity Score	6.82	4.41	< .001	5.68	6.18	.044	6.14	6.13	.997
	Mean Daily Number of News Reports	2.03	0.74	< .001	2.39	1.60	.007	2.14	1.50	.010

^*^ Each point of a Yahoo! search activity score equals 0.001% of the population searching Yahoo! on any day.

### Peaks in Yahoo! Cancer Search Activity and News Coverage

**Figure 1:**
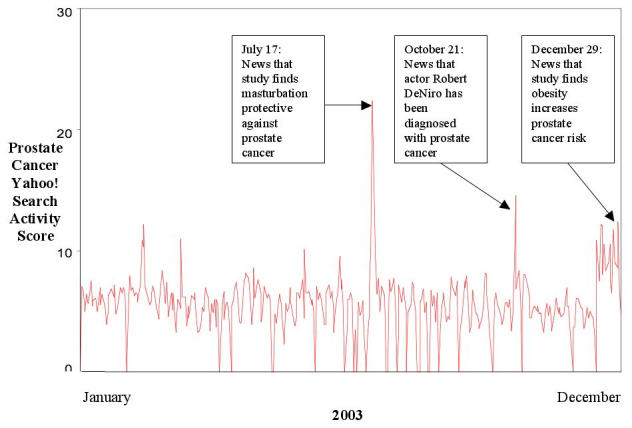
2003 US prostate cancer Yahoo! search activity (each point of a Yahoo! search activity score equals 0.001% of the population searching Yahoo! on any day)

On several occasions, Yahoo! search activity scores associated with breast cancer, colon cancer, and prostate cancer were marked by sharp increases of 100% or more from one day to the next. We investigated news events that corresponded with the highest three spikes in 2003 Yahoo! search activity for these cancers. These peaks in “breast cancer” and “colon cancer” search activity all occurred during their respective national awareness months and appeared to be related to news coverage promoting the awareness months. The highest peak in “prostate cancer” search activity (22.34) occurred on July 17 after news reports of a study [27] exploring the association between sexual behavior and prostate cancer risk (Figure 1). These news reports generally focused on the possible protective benefit of masturbation. This study was not covered widely by the US news media, but it generated substantial news coverage in Australia and filtered onto the Internet via chat rooms, message boards, and medical news Web pages. While there was no corresponding spike in “masturbation” search activity, there was a 117% increase in the search activity score (from 61.88 on July 16 to 133.08 on July 17) for “masterbation,” a common misspelling. The second highest spike in “prostate cancer” search activity (14.59) occurred on October 21 after news broke that Academy-Award-winning actor Robert DeNiro had been diagnosed with prostate cancer. This story was widely covered by the US news media, and a 277% increase in “Robert DeNiro” search activity was observed on the same date (from 15.87 on October 20 to 59.90 on October 21). The third highest peak in “prostate cancer” search activity (12.41) occurred on December 29, when a study linking obesity with increased prostate cancer risk [28] was covered by several US news outlets. No corresponding rise in searches for the terms “obesity,” “overweight,” or “weight loss” was observed.

## Discussion

This study suggests that media coverage plays a powerful role in prompting online cancer information seeking. News coverage correlated significantly with Yahoo! search activity (*P* < .001). Also, Yahoo! search activity was found to rise as news coverage increased, and sharp rises in search activity from one day to the next appeared to be associated with increases in relevant news coverage. This study also suggests that the Internet can rapidly disseminate health news: the highest spike in 2003 US “prostate cancer” Yahoo! search activity seemed to be generated largely by news coverage in Australia that rapidly filtered onto the Internet via chat rooms, message boards, and medical news Web pages. Thus, it possible that a news story does not necessarily have to be covered by the US news media in order to generate US Internet search activity.

News coverage volume also correlated with estimated cancer incidence (*P* = .015) and mortality (*P* < .001). This is interesting because past studies [29-33] on this topic have not generated consistent findings, with most [30-32] finding no relationship between disease burden and news coverage volume. However, none of the past studies focused on cancer, and none used our method for identifying news reports. While the news coverage of specific cancers generally matched their burden, we noted that some cancers, such as leukemia, ovarian cancer, and testicular cancer, were associated with more Internet search activity than their burden would suggest. A similar observation was reported by Bader and Theofanos [21], who suggested that this discrepancy may result from more searches being required to locate online information about less common cancers. The high correlation between cancer-specific news coverage and associated online search activity in the present study suggests another explanation: some cancers received a disproportionate share of news coverage relative to their incidence and mortality, and online search activity, often prompted by news coverage, reflects this imbalance.

We detected several periodicity effects in US Yahoo! cancer search activity, which tended to be higher on weekdays and during national cancer awareness months but lower during the summer months. It should be noted that these observations are not artifacts of the size of the online population during these periods because Yahoo! search activity scores are based on the percentage, not the number, of total users. One explanation for these results is that the volume of cancer news coverage tended to follow these trends. It is also possible that users tend to search for online cancer information from school or work settings. As a result, Yahoo! cancer search activity would be expected to drop during weekends when people are at home and over the summer months when many students are out of school and many workers go on vacation.

Although Yahoo! is a leading US Internet search engine, the extent to which the findings of this study can be generalized to other search engines is not known. Also, we were unable to discern the motivations of Yahoo! users searching for cancer information. For instance, news coverage of a breast cancer drug might be associated with an increase in “breast cancer” search activity. While the Yahoo! Buzz Index would detect this rise, it cannot tell how many searchers were breast cancer patients or family members and how many were investors interested in buying stock in the company developing the drug.

Internet search activity offers an innovative tool for passive surveillance of health information–seeking behavior. While our work focused on cancer, Internet search activity may be useful in gauging health information seeking related to other diseases. For example, the volume of Internet searches related to symptoms or conditions might be used to predict disease outbreaks (eg, influenza) or to assess mental health following a disaster. Researchers at the Centre for Global eHealth Innovation have begun to experiment with analyses of this type [34,35]. The Yahoo! Buzz Index is unique among Internet search datasets because it provides search activity scores adjusted for the size of the population searching for online information, which has steadily grown each year [19]. Perhaps in the future, other Internet search engines will offer databases similar to the Yahoo! Buzz Index, and research could be conducted using a combination of search engines.
